# Probiotic Supplementation Alleviates Corticosterone-Induced Fatty Liver Disease by Regulating Hepatic Lipogenesis and Increasing Gut Microbiota Diversity in Broilers

**DOI:** 10.3390/microorganisms13010200

**Published:** 2025-01-17

**Authors:** Yuyan Feng, Wenqing Mei, Qu Chen, Xiaojing Chen, Yingdong Ni, Mingming Lei, Jie Liu

**Affiliations:** 1 Institute of Animal Husbandry, Jiangsu Academy of Agricultural Sciences, Nanjing 210094, China; fyy@stu.njau.edu.cn (Y.F.); cxj@stu.njau.edu.cn (X.C.); 2 Key Laboratory of Animal Physiologic and Biochemistry, College of Veterinary Medicine, Ministry of Agriculture and Rural Affairs, Nanjing Agricultural University, Nanjing 210094, China; mwq@stu.njau.edu.cn (W.M.); 2018207007@njau.edu.cn (Q.C.); niyingdong@njau.edu.cn (Y.N.)

**Keywords:** fatty liver disease, corticosterone, probiotic, gut microbiota, broiler

## Abstract

Emerging evidence indicates a close relationship between gut microbiota and fatty liver disease. It has been suggested that gut microbiota modulation with probiotics ameliorates fatty liver disease in rodents and humans, yet it remains unclear whether the same results will also be obtained in poultry. The aim of this study was to investigate whether a mixture of probiotics supplemented after hatching can prevent CORT-induced fatty liver disease in broilers, and to determine how such effects, if any, are associated with hepatic de novo lipogenesis and gut microbiota composition. Ninety-six one-day-old green-legged chickens were divided into a control group (CON) and probiotic group (PB). At 28 days of age, fatty liver was induced in 16 broilers that were randomly selected from the CON or PB group. At the end of the experiment, broilers from four groups, (i) the control group (CON), (ii) corticosterone group (CORT), (iii) probiotic group (PB), and (iv) PB plus CORT group (CORT&PB), were slaughtered for sampling and analysis. The results showed that probiotic administration significantly prevented CORT-induced body weight loss (*p* < 0.05) but did not alleviate the weight loss of immune organs caused by CORT. Compared to CON, the broilers in the CORT group exhibited a significant increase in triglyceride (TG) levels in plasma and liver (*p* < 0.01), as well as severe hepatocytic steatosis and hepatocellular ballooning, which was accompanied by the upregulation of hepatic lipogenesis gene expression. However, probiotic supplementation markedly decreased the intrahepatic lipid accumulation and steatosis histological score, which was associated with the downregulation of sterol regulatory element-binding protein-1 (*SREBP1*) and acetyl-CoA carboxylase (*ACC*) mRNA (*p* < 0.05) and the expression of its protein (*p* = 0.06). The cecal microbiota composition was determined by 16S rRNA high-throughput sequencing. The results showed that CORT treatment induced distinct gut microbiota alterations with a decrease in microbial diversity and an increase in *Proteobacteria* abundance (*p* < 0.05). In contrast, probiotic supplementation increased the beta diversity, the community richness, and the diversity index (*p* > 0.05), as well as the abundance of *Intestinimonas* (*p* < 0.05). Our results indicate that CORT treatment induced severe fatty liver disease and altered the gut microbiota composition in broilers. However, post-hatching probiotic supplementation had a beneficial effect on alleviating fatty liver disease by regulating lipogenic gene expression and increasing gut microbiota diversity and the abundance of beneficial bacteria. We demonstrate for the first time that the supplementation of probiotics to chicks had a beneficial effect on preventing fatty liver disease through regulating lipogenic gene expression and improving the gut microbial balance. Thus, our results indicate that probiotics are a potential nutritional agent for preventing fatty liver disease in chickens.

## 1. Introduction

Nonalcoholic fatty liver disease (NAFLD) is a burgeoning health problem that affects a considerable proportion of adults in Western countries as well as developing countries [1]. It comprises a spectrum of liver pathology including hepatocellular steatosis, steatohepatitis, fibrosis, and cirrhosis [2]. In chickens, the relatively basic lymphatic system and transport of fat in chylomicrons into the portal vein increase their susceptibility to hepatic fat deposition. The accumulation of TG in the liver was the main feature of fatty liver disease [3]. Hepatic de novo lipogenesis (DNL) is thought to play a pivotal role in the development of fatty liver disease [4]. The key enzymes in the process of DNL include sterol regulatory element-binding protein-1 (*SREBP1*), fatty acid synthase (*FASN*), and acetyl-CoA carboxylase (*ACC*). Thus, lipogenesis has become a target for the prevention and therapeutic treatment of fatty liver disease.

Glucocorticoid hormones (GCs) are important regulators of lipid metabolism, promoting lipogenesis when treated with chronic exposure [5]. For instance, chronic excessive GC exposure induced whole-body insulin resistance, strongly related to metabolic dysfunction including intrahepatic lipid accumulation, intramuscular fat deposition, and abdominal adiposity in humans [6,7]. Corticosterone (CORT) is the main active form of GC in chickens. Previous studies have shown that fatty liver disease can be induced by excessive CORT exposure in broilers [8,9,10].

The relationship between intestinal microbiota dysbiosis and liver diseases has been reported [11]. Intestinal microbiota has been proposed as a regulator of energy homeostasis and ectopic fat deposition, which indicates its implications in metabolic diseases [12]. Thus, the change in gut microbiota seems to play an important role in the induction and promotion of liver injury. Although the mechanism of probiotics is not yet fully understood, it has been considered to be the possible adjunctive therapy in fatty liver disease due to the numerous beneficial effects such as improving epithelial barrier function, preventing bacterial translocation, inhibiting bacterial mucosal adhesion, and reducing inflammation [12]. Probiotic supplementation can prevent the occurrence and development of fatty liver disease, and improve liver steatosis and fibrosis in mice fed with a high-fat diet (HFD) [13,14]. The protective effects of probiotics on fatty liver disease by many pathways including reducing hepatic lipid deposition, endotoxemia, and oxidative stress [15]. It is reported that *Parabacteroides distasonis* can alleviate the disorder of lipid metabolism dysfunction in ob/ob mice and HFD mice, reduce hyperglycemia and hyperlipidemia, and improve liver steatosis [16]. However, the effect of the supplementation of probiotics complex during the early growth stage of broilers on the gut microbiota profiles and the alleviating effect on fatty liver disease in broilers still waits for further investigation.

Therefore, this study induced the fatty liver of broiler chickens by CORT and added probiotic complex after hatching can alleviate fatty liver and further explored the relationship between liver new fat generation and intestinal microbiota composition. The results of this study provide reference and research information for probiotics to promote the development of animal husbandry based on clarifying that probiotics are beneficial to intestinal health.

## 2. Materials and Methods

### 2.1. Ethics Approval and Consent to Participate

The Animal Management and Ethics Committee of Nanjing Agricultural University (IACUC) approved all animal procedures. Sampling and slaughter procedures were in accordance with “Guidelines on Ethical Treatment of Experimental Animals” (2006) No. 398 set by the Ministry of Science and Technology, China and “Regulation Regarding the Management and Treatment of Experimental Animals” (2008) No. 45 formulated by the Jiangsu Provincial People’s Government.

### 2.2. Animals and Treatment

A total of 96 one-day-old green-legged chickens were purchased from the Zhenjiang Wenshi company. During the experiment, the hens were fed a basal diet and were given free access to water. The ambient temperature was maintained at 35~37 °C during the first 3 days, then gradually decreased by 0.5 °C every day until reaching a final temperature of 21 °C. Ninety-six one-day-old green-legged chicken were divided into a control group (CON) and probiotic group (PB), respectively. Dietary composition is shown in Table 1. Broilers were randomly divided into a control (CON) group and probiotic (PB) group. Each group contains 48 broilers, divided into 6 cages (length 45 cm, width 57 cm, height 40 cm) with 8 chickens in each cage. PB group chickens received probiotics of 100 mg per chicken, respectively, via drinking water (boiled water below 30 °C) for two weeks, and drinking water was changed every day. At 28 days of age, broilers were randomly divided into 4 groups (8 broilers per group): control group (CON), corticosterone group (CORT), probiotic group (PB), PB and CORT treated group (CORT&PB) (16 broilers were randomly selected from the CON group and PB group). Induced fatty liver by subcutaneous injection of CORT (4.0 mg/kg) daily for 1 week. Body weight was recorded from 1 to 35 days of age. Thirty-two broilers were slaughtered at 36 days of age, with eight broilers in each group. The immune organs of the liver, spleen, bursa of fabricius, and thymus were weighed. Blood samples were collected and centrifuged at 3500 rpm for 10 min, and plasma samples were separated and stored at −20 °C. Subsequently, liver samples were collected into liquid nitrogen and stored at −80 °C for further analysis. The liver samples used to make the tissue sections were placed in 4% paraformaldehyde. The cecal contents were collected and stored at −20 °C. The project has been approved by the Animal Protection and Utilization Committee of Nanjing Agricultural University (NJAU-2021-008).

During the test, incandescent lamps (warm yellow) were used in the coop, the light intensity was about 20 lux, and the light intensity gradually decreased with the increase in day age. During feeding, the relative humidity in the chicken house is maintained at 50–65%. At room temperature, the microorganisms in probiotics can survive for 5–20 days. When the drinking water temperature is too high, the bacteria will be inactivated. Probiotics, purchased from Jiangsu H.F.Q. Technology Co., Ltd. (Taizhou, China), are composed of *Bifidobacterium*, *Lactobacillus acidophilus*, *Streptococcus faecalis*, and *yeast*. *Bifidobacterium* and *Lactobacillus acidophilus* were not less than 1.0 × 10^7^ CFU, and *Streptococcus faecalis* and *yeast* were not less than 1.0 × 10^6^ CFU per gram. Corticosterone (C_25_O_5_) were purchased from Sigma-Aldrich Chemical Co. (St. Louis, MO, USA).

### 2.3. Histopathology

Liver specimens were fixed with 4% formaldehyde-buffered solution and paraffin-embedded and then sectioned for hematoxylin eosin (HE) staining. Frozen sections of the liver were stained with Oil Red. Samples were rated based on the severity of steatosis and graded 0–4, indicating none, slight, mild, moderate, and severe, respectively. Steatosis scores were analyzed independently by three veterinarians with reference to a previous publication [8]. In each case, five tissue sections were examined, and a numerical score was assigned.

### 2.4. Plasma Biochemical Indicators Measurement and TG Concentration in Liver

The concentration of triglyceride (TG) in plasma was measured by an automatic biochemical analyzer (7020, HITACHI, Tokyo, Japan) using commercial kits (E1003; Applygen Technologies Inc., Beijing, China). Hepatic TG was measured using a triglyceride assay kit (E1013; Applygen Technologies Inc., Beijing, China) following the manufacturer’s instructions.

### 2.5. Reverse Transcription and Real-Time PCR

Total RNA was isolated from liver samples with the trizol reagent (15596026; Invitrogen, Shanghai, China). The concentration and quality of the RNA were assessed by a Nano Drop ND-1000 Spectrophotometer (Thermo Fisher Scientific, Madison, WI, USA). Next, 2 μg of total RNA was treated with RNase-Free DNase (M6101; Promega, Madison, WI, USA) and was reverse transcribed according to the manufacturer’s instructions. Two microliters of diluted cDNA (1:20, *v*/*v*) were used for real-time PCR with a Mx3000 P Real-Time PCR System (Stratagene, La Jolla, CA, USA). *β-actin*, which is not affected by CORT and PB, was chosen as the reference gene. Primer sequences of internal reference gene *β-actin* and target gene were designed using Primer Premier 5.0 software. Synthesized by Nanjing Tsingke Biotechnology Co., Ltd. (Nanjing, China). The primer sequence is shown in Table 2. The 2^−ΔΔCT^ method was used to analyze the real-time PCR data [17].

### 2.6. Western Blotting Analysis

The total protein was extracted from a 30 mg sample of frozen liver as previously described [18]. A BCA Protein Assay kit (Pierce, Rockford, IL, USA) was used to measure the protein concentration. In total, 40 µg sample of protein was separated by 10% SDS-PAGE gel. Western blot analysis for *FASN* (AB22759, Abcam, 1:500, Cambridge, UK), *ACC* (AF6421, Affinity, 1:500, Ancaster, ON, Canada), CPT1A (ab83862, Abcam, 1:200), and CPT1B (AB134998, Abcam, 1:500) were carried out following the manufacturer’s instructions. Images were captured by VersaDoc 4000MP system (Bio-Rad, Hercules, CA, USA) and the band density was analyzed with image j (National Institutes of Health, NIH, Bethesda, MD, USA).

### 2.7. Extraction of Genome DNA and 16S rRNA Sequencing

Total genome DNA from samples was extracted using the CTAB/SDS method. DNA concentration and purity were monitored on 1% agarose gels. According to the concentration, DNA was diluted to 1 ng/µL using sterile water. The 16S rRNA genes were amplified in distinct regions (16S V3-V4) using specific primers with barcodes. Phusion ^®^ high-fidelity PCR master mixed with GC buffer from New England Biolabs and high-fidelity enzyme were used for PCR to ensure the efficiency and accuracy of amplification. The PCR product was verified by electrophoresis in 2% agarose gel and then recycled by the GeneJET^TM^ Gel Extraction Kit (Thermo Scientific, Waltham, MA, USA). Sequencing libraries were generated using Ion Plus Fragment Library Kit 48 rxns (Thermo Scientific) following the manufacturer’s instructions and sequenced on an Ion S5^TM^ XL platform. Sequences analysis was performed by Uparse software. Species annotation analysis was measured using the mother method and the Silva Database. Data were normalized using a standard of sequence number according to the least sequences. Alpha and Beta diversity was calculated and displayed with QIIME (Version1.7.0) and R software (Version 2.15.3). The above experiments were conducted by Novogene Co., Ltd. (Beijing, China) completed.

### 2.8. Statistical Analysis

Results are expressed as means ± SEM and were analyzed by two-way ANOVA using the General Linear Model GLM) producer in SPSS 20.0 for Windows (IBM Cooperation, Armonk, NY, USA). Data were analyzed using a 2 × 2 factorial arrangement with the main effects of CORT and PB treatment. LSD post hoc analysis was used for evaluating differences among specific groups. The level of significance was based on the probability value *p* < 0.05.

## 3. Results

### 3.1. Body Weight and the Relative Weight of Immune Organs

**Figure 1:**
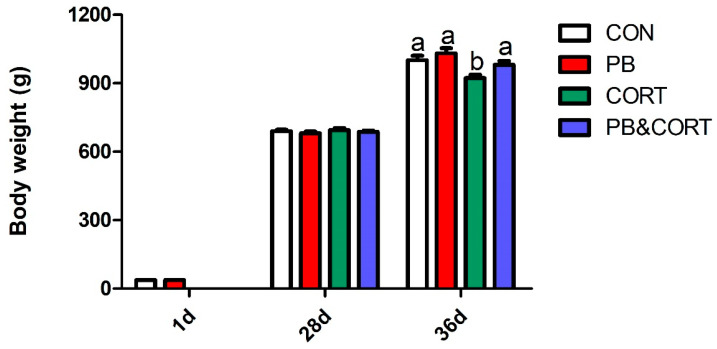
Effect of probiotic and CORT on body weight of broilers. CON, control group; PB, probiotic-treated group; CORT, corticosterone-treated group; PB&CORT, PB and CORT-treated group. Data are expressed as means ± SEM (*n* = 8). Different small letter superscripts a, b indicates significant differences (*p* < 0.05).

At 28 days of age, there was no significant difference in body weight between the CON and PB groups (*p* > 0.05; Figure 1). Compared to CON, CORT administration markedly decreased body weight (*p* < 0.01; Figure 1); however, chickens in the CORT&PB group showed higher body weight than those in the CORT group at 36 days of age (*p* < 0.05; Figure 1).

Compared with the CON group, CORT treatment significantly increased the liver weight and index (*p* < 0.01; Table 3), while PB supplementation significantly decreased the liver index (*p* < 0.05; Table 3). The weight of immune organs including thymus, spleen, and bursa of fabricius and their relative weight were significantly decreased by CORT injection compared to CON (*p* < 0.01; Table 3). PB supplementation did not alter the immune organs growth (*p* > 0.05; Table 3).

### 3.2. Hepatic Histological Analysis

**Figure 2:**
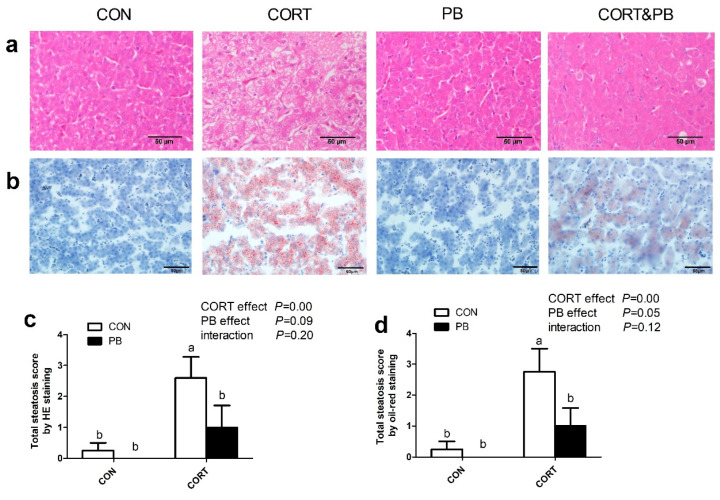
Effect of probiotic and CORT on hepatic hematoxylin-eosin (HE) staining (×200) and oil-red (×400) staining of broilers. (**a**), HE stains; (**b**), Oil-red staining; (**c**), total steatosis score by HE staining; (**d**), total steatosis score by oil-red staining. CON, control group; CORT, corticosterone-treated group; PB, probiotic-treated group; CORT&PB, CORT and PB-treated group. Data are expressed as means ± SEM (*n* = 5). Different small letter superscripts a, b indicates significant differences (*p* < 0.05).

Hepatic histological sections stained with hematoxylin-eosin (HE) and Oil Red O showed that severe hepatic lipid accumulation was obviously observed in the CORT group; however, only slight vacuolar steatosis was shown in the CORT&PB group (Figure 2a,b). As shown in Figure 2c,d, CORT injection significantly increased the total steatosis score estimated by the HE stains and Oil Red O staining (*p* < 0.05), yet PB treatment tended to decrease it (*p* = 0.09).

### 3.3. TG Concentrations in Plasma and the Liver

**Figure 3:**
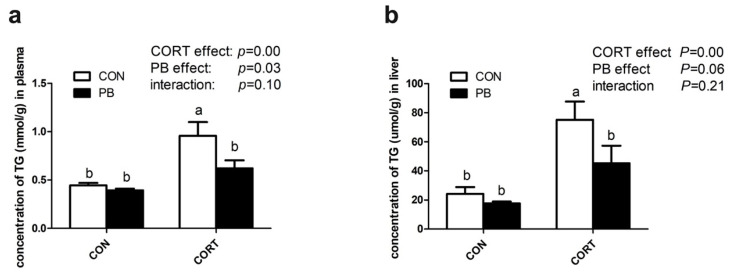
TG content in plasma and liver. (**a**), TG content in plasma; (**b**), TG content in plasma. C-CON, control group; C-CORT, corticosterone-treated group; P-CON, PB-treated group; P-CORT, PB- and CORT-treated group. Data are expressed as means ± SEM (*n* = 5). Different small letter superscripts a, b in a row indicates significant differences (*p* < 0.05).

As shown in Figure 3, CORT treatment significantly increased the TG concentration in plasma and the liver compared to the control group (*p* < 0.01), and PB administration significantly decreased plasma TG concentration (*p* < 0.05) and tended to reduce the hepatic TG level (*p* = 0.06). The CORT&PB group showed lower levels of TG in plasma and the liver when compared to the CORT group (*p* < 0.05).

### 3.4. Expression of Genes Related to TG Metabolism in the Liver

**Figure 4:**
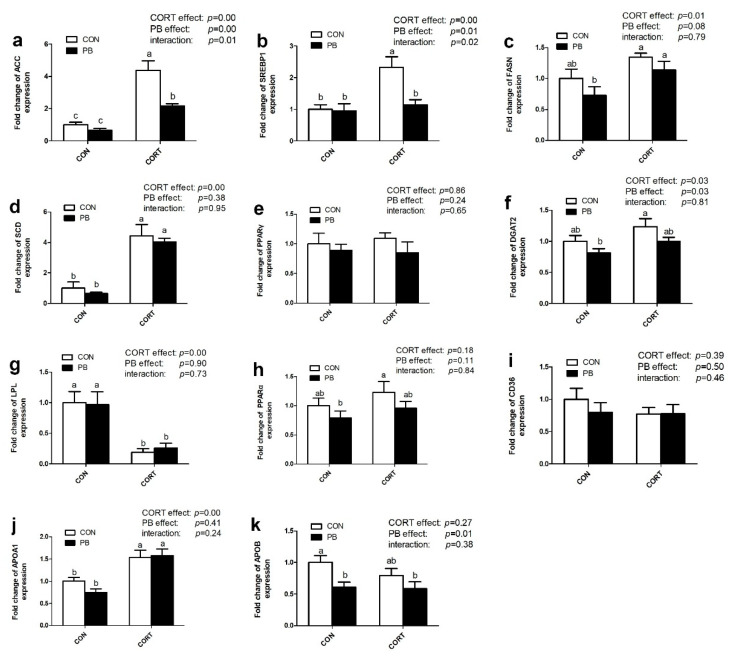
Effect of probiotic and CORT on liver expression of genes related to lipogenesis. (**a**), Hepatic *ACC* mRNA expression; (**b**), Hepatic *SREBP1* mRNA expression; (**c**), Hepatic *FASN* mRNA expression; (**d**), Hepatic *SCD* mRNA expression; (**e**), Hepatic PPARα mRNA expression; (**f**), Hepatic *DGAT2* mRNA expression; (**g**), Hepatic *LPL* mRNA expression; (**h**), Hepatic PPARα mRNA expression; (**i**), Hepatic *CD36* mRNA expression; (**j**), Hepatic *APOA1* mRNA expression; (**k**), Hepatic *APOB* mRNA expression; C-CON, control group; C-CORT, corticosterone-treated group; P-CON, PB-treated group; P-CORT, PB- and CORT-treated group. Data are expressed as means ± SEM (*n* = 8). Different small letter superscripts a–c indicates significant differences (*p* < 0.05).

As shown in Figure 4, CORT significantly up-regulated hepatic *ACC* (*p* < 0.01), *SREBP1* (*p* < 0.01), *FASN* (*p* < 0.05), *SCD* (*p* < 0.01), and *DGAT2* (*p* < 0.05) mRNA expression; however, PB treatment markedly down-regulated *ACC* (*p* < 0.01), *SREBP1* (*p* < 0.05), *DGAT2* (*p* < 0.05) and *FASN* (*p* = 0.08) mRNA expression in the liver. Moreover, there was a significant interaction of CORT and PB on hepatic *ACC* and *SREBP1* genes expression (*p* < 0.05) (Figure 4a,b).

The expression of genes related to lipolysis and TG transport in the liver was also changed by CORT but not PB treatment. CORT significantly down-regulated *LPL* mRNA expression (*p* < 0.01) but not *PPARα* expression (*p* > 0.05). There was no significant effect of PB on these two genes expression *(p* > 0.05; Figure 4g,h). CORT significantly up-regulated hepatic *APOA1* mRNA expression (*p* < 0.01; Figure 4j) but did not change *CD36* or *APOB* mRNA expression (*p* > 0.05; Figure 4i,k). Probiotic significantly down-regulated hepatic *APOB* mRNA expression (*p* < 0.05; Figure 4k).

### 3.5. Expression of Protein Related to Lipid Metabolism in the Liver

**Figure 5:**
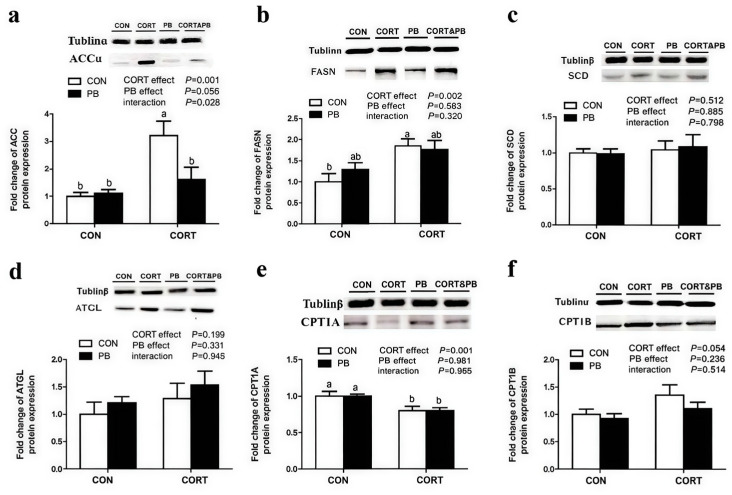
Effect of probiotic and CORT on liver expression of genes related to lipolysis. (**a**), Hepatic *ACC* protein expression; (**b**), Hepatic *FASN* mRNA expression; (**c**), Hepatic *SCD* protein expression; (**d**), Hepatic ATGL mRNA expression; (**e**), Hepatic CPT1A mRNA expression; (**f**), Hepatic CPT1B mRNA expression; C-CON, control group; C-CORT, corticosterone-treated group; P-CON, PB-treated group; P-CORT, PB- and CORT-treated group. Data are expressed as means ± SEM (*n* = 4). Different small letter superscripts a, b indicates significant differences (*p* < 0.05).

As shown in Figure 5, CORT treatment significantly decreased the protein expression of CPT1A in liver (*p* < 0.01) but significantly increased the protein expression level of CPT1B (*p* < 0.05). CORT and PB treatment had no significant effect on the expression of *SCD* and ATGL protein (*p* > 0.05). CORT treatment significantly increased the protein expressions of *FASN* and *ACC* in liver (*p* < 0.01), PB treatment had no significant effect on *FASN*, but tended to decrease the expression of *ACC* protein, and PB and CORT had a significant interaction effect on *ACC* protein expression (0.05 < *p* < 0.1).

### 3.6. The Analysis of Cecum Microbiota Composition

**Figure 6:**
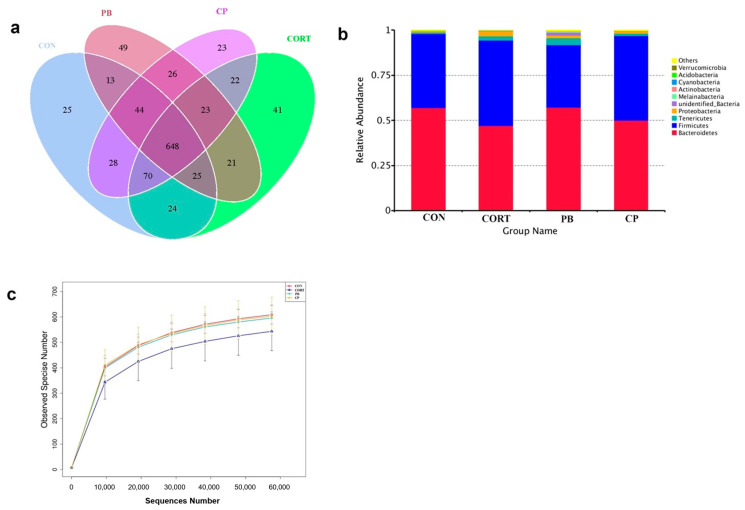
(**a**), Venn Graph; (**b**), Relative abundance of species at phylum level; (**c**), Rarefaction Curve; CP, CORT and PB- treated group. *n* = 4.

A total of 1,227,484 clean reads were obtained for the bacterial 16S rRNA genes by high-throughput sequencing analysis and 1082 OUTs were obtained by clustering at 97% identity. The number of common and unique OTUs among four groups was shown in the Venn diagram (Figure 6a), describing sample similarity and overlap intuitively. The PB group had the highest number of unique sequences (49 OTUs), followed by the CORT group (41 OTUs), the CON group (25 OTUs), and the CORT&PB group (23 OTUs) (Figure 6a). In addition, there were 648 OTUs (approximately 60% of total OTUs) shared among four groups (Figure 6a). A rarefaction curve was performed by the number of OTUs, and sequences randomly extracted from samples, directly reflecting the rationality of sequencing data. Figure 6c shows that as the depth of sequencing increases, the curve tends to be flat, indicating that the sequencing results were reasonable, and each sample can truly reflect the microbial composition.

At the phylum level, *Firmicutes*, *Proteobacteria*, and *Bacteroidetes* were the most prominent in four groups (Figure 6b). We found an obvious phylum-wide shift in the *Proteobacteria* induced by CORT challenge (Figure 6b). *Proteobacteria* abundance was significantly increased in the CORT group compared with the CON group (Figure 6b).

**Figure 7:**
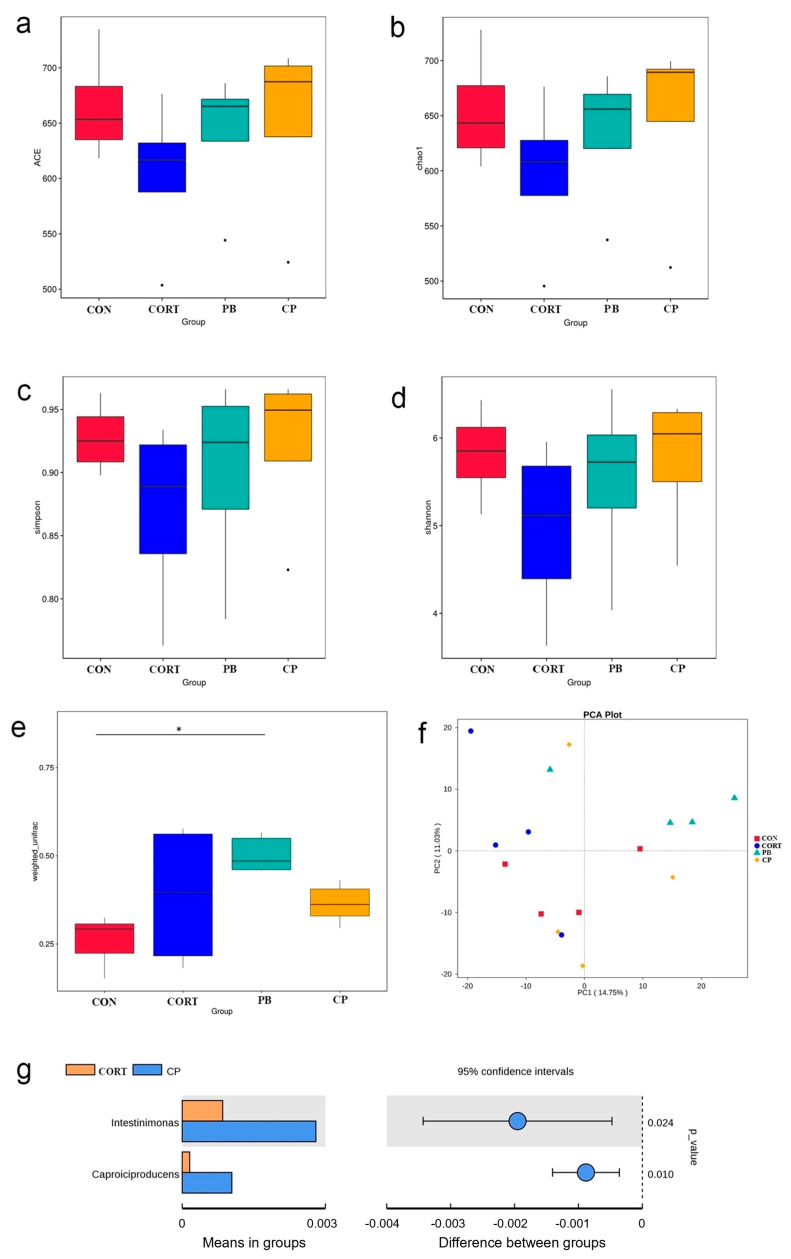
Alpha and Beta diversity analysis. The bacterial richness is estimated by ACE index (**a**) and Chao1 index (**b**); The bacterial diversity estimated by the Shannon index (**c**) and Simpson index (**d**). (**e**), Beta diversity based on weighted unifrac; (**f**), Principal Component Analysis (PCA); (**g**), Differences species analysis by *t*-test. CP, CORT- and PB-treated group *n* = 4.

The community richness and diversity of cecal microbiota were presented by the Chao index, ACE index, Shannon index, and Simpson index. CORT treatment reduces the four indices, yet this effect was reversed by PB supplementation (*p* > 0.05; Figure 7a–d). Compared with the CON group, broilers in PB group had higher beta diversity based on weighted unifrac (*p* < 0.05; Figure 7e). The community composition of gut microbiota was analyzed by principal component analysis (PCA). We found that PCA can notably show significant differences in cecal microbiota composition between four groups (Figure 7f). The PCA score plot showed that the gut microbiota of the CORT group exhibited a shift along the positive direction of the second principal component (PC2) compared with that of the CON group, and the gut microbiota of the PB group showed a notable shift along the positive direction of the PC2 as well as the positive direction of the first principal component (PC1) (Figure 7f). Interestingly, bacterial composition in the CORT&PB group was like that in the CON group. Collectively, the community composition of gut microbiota altered by the CORT challenge was partly reversed by probiotics administration tracing to be like the profile of the control chickens.

Furthermore, the differences between CORT and CORT&PB group in genus level were also analyzed by *t*-test. Compared to the CORT group, the abundance of *Intestinimonas* and *Caproiciproducens* was significantly increased in CORT&PB group (Figure 7g).

## 4. Discussion

Fatty liver disease becomes a concerning health problem in domestic chickens, causing a significant economic loss in the poultry industry. The liver is a vital organ for lipid metabolism, particularly in the avian species. Lipid drops usually accumulate in liver tissue, causing hepatic structural disruption and dysfunction, which ultimately leads to liver hypertrophy and fatty liver disease [19]. Therefore, controlling lipid accumulation in the liver can prevent the progression of lipid metabolic disorders [20]. In this study, fatty liver disease was mimicked in broilers by the consecutive injection of CORT, showing the typical characteristics of fatty liver disease as the previous report [10]. However, our results showed that probiotics supplementation from post-hatching for 2 weeks could effectively protect the loss of body weight and prevent the progress of fatty liver disease induced by CORT in broilers. It is reported that chronic CORT treatment reduces protein synthesis but promotes protein degradation, and finally leading to the loss of body weight in chickens [10,21]. It has been described that the probiotic exhibits a beneficial effect on body weight gain [22,23]. Wang et al. [24] found that *Bacillus amyloliquefaciens* SC06 administration improves HFD-induced poor growth performance. Our results are consistent with the above research.

The significant increase in TG concentration in plasma and liver, as well as histological features including steatosis and vacuolar degeneration by CORT injection in the present study was in accordance with our previous work in chickens subjected to exogenous CORT exposure [9] and in rats exposed to dexamethasone (DEX) challenges [25]. Interestingly, our results clearly showed that these phenotypic characterizations of fatty liver disease in broilers were greatly ameliorated by the administration of probiotic mixture administration. Consistent with the results of this study, Wang et al. [16] reported that *Parabacteroides distasonis* alleviates an increase in intrahepatic triglyceride in high-fat diet (HFD)-fed mice. Yalçin et al. [26] also obtained similar results, indicating that the supplementation of probiotics to broilers play a beneficial effect on alleviating fatty liver disease. In chickens, fatty acid synthesis primarily occurs in the liver, whereas in mammals, adipose tissue is the main lipogenic tissue [27]. Four major pathways control hepatic TG accumulation: TG import via up-taking the dietary TG, de novo lipogenesis, the utilization of TG through fatty acid β-oxidation (lipolysis), and TG export from the liver [28]. Many studies have confirmed that de novo lipogenesis plays a vital role in the progression of fatty liver disease [29]. The previous study indicated that hepatic fat deposition in NAFLD patients is mainly attributed to the highly regulated metabolic pathway for the synthesis of fatty acids from *ACC* [29]. Consistently, the fatty liver disease caused by exogenous glucocorticoids exposure mainly resulted from the activation of lipogenesis in chickens [9] as well as in rats [25]. In this study, our results indicated that the mRNA expression levels of *ACC*, the key rate-limiting enzyme of de novo lipogenesis, and SREBP-1, a key transcription factor downstream of *ACC*, in the chicken liver were increased by CORT challenges. Notably, probiotic supplementation could alleviate the CORT challenge-mediated activation of the gene expression of lipogenesis in birds. Similar results have been reported by Wang et al. [30] who noted that *Lactobacillus johnsonii* BS15 treatment could improve lipid metabolism in the liver by inhibiting the hepatic fatty acid de novo synthesis, accompanied by down-regulated SREBP-1 and its target genes, *FASN* and *SCD*. In addition, although CORT treatment had significant effects on lipolysis and lipid transport, probiotics supplementation has little effect. These results present convincing evidence to support our view that the possible mechanism by which probiotics protect fatty liver disease induced by CORT injection may be associated with diminished gene expression levels of the DNL pathway.

Modulation of gut microbiota with probiotics have recently been implemented in the prevention and treatment of several metabolic diseases, such as fatty liver disease [31]. Hamid et al. also confirmed that general microbiota imbalance was linked with NAFLD [32]. Probiotics are considered to normalize gut microbiota and reverse microbial dysbiosis, which could potentially benefit hosts. Ritze reported that *Lactobacillus rhamnosus GG* attenuated liver inflammation and steatosis in high-glucose diet-induced NAFLD model mice [33]. A study showed that dietary supplementation with a probiotic, the Primalac 454, was performed in 3-week-old Ross-308 broiler chickens for 4 weeks, which can limit the intensity of the liver fatty disease induced with dietary protein restriction (14% vs. 20% crude protein) [26]. Wang et al. [30] reported that dietary supplementation of *Lactobacillus johnsonii* BS15 improves hepatic lipid metabolism in broilers, accompanied by the lower *Firmicutes*/*Bacteroidetes* ratio. A low *Firmicutes*/*Bacteroidetes* ratio can be effective against obesity [34]. Here, we indicate that the composition of the gut microbiota seems to play a role in the prevention of fatty liver disease. We showed that the richness and diversity of the community decreased in broilers with CORT-induced fatty liver disease. Similar results have been observed in the studies of subjects suffering from the illness, such as obesity [35] and liver cirrhosis [36]. Interestingly, an increase in alpha and beta diversity of gut microbiota was observed after probiotic supplementation, and it was similar to the previous results of Zhou et al. [37], who reported that probiotic treatment could increase the Shannon index in HFD rats. Furthermore, we also observed a significant alteration of gut microbiota under the condition of CORT injection. The result of alterations in gut microbiota composition induced by CORT injection increases the possibilities of microbial dysbiosis, which may play a part in increased susceptibility to fatty liver diseases. However, it is demonstrated that the probiotic prevented diminished bacterial diversity and severe gut microbiota alteration, which is consistent with Zhou et al. [37] who reported that probiotic treatment could prevent NAFLD progression via balance in the gut microbiota. In the present study, the abundance of *Proteobacteria* was markedly enhanced by CORT exposure, and a similar result has been reported by Hamid et al. [33], who noted that non-alcoholic steatohepatitis (NASH) subjects exhibited an increased abundance of *Proteobacteria* in laying hens. A study has shown that more abundance of *Proteobacteria* was found in the fecal matter of children who were high fat and low fiber consumers [38]. Notably, *Intestinimonas*, a short-chain fatty acid (SCFA)-producing intestinal bacterium [22], was enriched in the CORT&PB group. It has been reported that SCFA is generally considered to have many important effects on maintaining host health such as providing nutrients and energy for the host [39], and the reduced production of SCFA is a crucial pathogenic cause of fatty liver disease [40]. Our findings were consistent with these studies, indicating that probiotic supplementation could enrich the relative abundance of SCFA producers and change the proportion of the gut microbiota [41,42].

## 5. Conclusions

We demonstrate for the first time that the supplementation of probiotics to chicks had a beneficial effect on preventing fatty liver disease through regulating lipogenic gene expression and improving gut microbial balance. Thus, our results indicate that probiotics could represent a potential nutritional agent for preventing fatty liver disease in chickens.

## Figures and Tables

**Table 1:** Ingredients and nutrient composition of diets.

Ingredient %	Content	Composition	Content
Corn	57.61	ME (MJ/kg)	12.56
Soybean meal	31.00	CP (g/kg)	211.00
Corn protein powder	3.29	Ca (g/kg)	10.00
Soybean oil	3.11	P (g/kg)	4.60
Limestone	1.20	Lys (g/kg)	12.00
Dicalcium P	2.00	Met (g/kg)	5.00
L- Lysine	0.34	Met + Cys (g/kg)	8.50
DL-Met	0.15		
NaCl	0.30		
Premix ^1^	1.00		

^1^ Premix is supplied per kilogram of diet: vitamin A 3 mg, vitamin D3 0.0075 mg, vitamin E 30 mg, vitamin K3 1.3 mg, thiamine 2.2 mg, riboflavin 8 mg, niacin 40 mg, calcium pantothenate 10 mg, pyridoxine 4 mg, folic acid 1 mg, biotin 0.04 mg, vitamin B12 0.013 mg, choline chloride 600 mg, iron 80 mg, zinc 60 mg, manganese 110 mg, copper 8.0 mg, iodine 1.1 mg, and selenium 0.3 mg.

**Table 2:** PCR primers sequence.

Genes	Genbank Accession	Primer Sequences (5′ to 3′)	Fragment Size
*ACC*	NM_205505.1	F: TGTGGGCTTTAGGAGATAA	136
		R: GGAACATTCAGGATACGC	
*APOA1*	NM_205525.4	F: ATGCCATCGCCCAGTTCG	165
		R: GCAGAGCCTCGGTGTCCTT	
*APOB*	NM_001044633.1	F: CAGAGGTAGAGGCAGGAC	82
		R: TCATCGGAGAAGTTAGGA	
*CD36*	NM_001030731.1	F: GCATCATTTCCTCCATTT	110
		R: ATTCCCTTCACGGTCTTA	
*DGAT2*	XM_419374.6	F: GCTCTTCTCCTCGAACACG	184
		R: CAACCCGAACCTGCCTTT	
*FASN*	NM_205155.3	F: GGGAATGTCACACCTTGCTC	164
		R: GGAAATGGGTATTGTCGCTC	
*LPL*	NM_205282.1	F: CGGTGACAGGAATGTATGA	140
		R: CTTCGTGTAAGCAGCAGA	
*PPAR-α*	AF163809.1	F: TTGTCGCTGCCATCATTT	147
		R: GAAGTTTCGGGAAGAGGA	
*PPAR-γ*	NM_001001460.1	F: CAGTGCAGGAGATTACAG	87
		R: CATATTTCAGGAGGGTTA	
*SCD*	NM_204890.1	F: GTTTCCACAACTACCACCAT	173
		R: ATCTCCAGTCCGCATTTT	
*SREBP1*	NM_204126.2	F: GGCAGAGGAAGACAAAGGC	123
		R: AGCAGCAGTGACTCCGAGC	
*β-actin*	L08165.1	F: CCCTGTATGCCTCTGGTC	194
		R: CTCGGCTGTGGTGGTGAA	

*ACC*, acetyl-CoA carboxylase; *APOA1*, Apolipoprotein A1; *APOB*, Apolipoprotein B; *CD36*, *CD36* molecule; *DGAT2*, diacylglycerol O-Acyltransferase 2; *FASN*, fatty acid synthase; *LPL*, lipoprotein lipase; *PPAR-α*, peroxisome proliferator-activated receptor alpha; *PPAR-γ*, peroxisome proliferator-activated receptor gamma; *SCD*, stearoyl-CoA desaturase; *SREBP1*, sterol regulatory element-binding protein-1.

**Table 3:** Effect of probiotics and CORT on organs weight and indexes of broilers.

Parameters	CON	CORT	PB	CORT&PB	*p*-Value		
					CT	PB	Interaction
**Liver weight (g)**	26.37 ± 0.65 ^b^	32.71 ± 1.83 ^a^	23.71 ± 1.11 ^b^	32.16 ± 1.78 ^a^	0.00	0.27	0.47
**Liver index (%)**	2.72 ± 0.05 ^b^	3.54 ± 0.17 ^a^	2.37 ± 0.07 ^b^	3.28 ± 0.16 ^a^	0.00	0.02	0.74
**Thymus weight(g)**	3.58 ± 0.24 ^b^	0.91 ± 0.07 ^a^	3.92 ± 0.52 ^b^	1.30 ± 0.12 ^a^	0.00	0.25	0.93
**Thymus index (%)**	0.36 ± 0.03 ^b^	0.11 ± 0.01 ^a^	0.39 ± 0.04 ^b^	0.13 ± 0.01 ^a^	0.00	0.32	0.95
**Spleen weight (g)**	1.84 ± 0.09 ^ac^	1.28 ± 0.09 ^b^	2.07 ± 0.20 ^a^	1.55 ± 0.17 ^bc^	0.00	0.11	0.88
**Spleen index (%)**	0.18 ± 0.01 ^ac^	0.14 ± 0.01 ^b^	0.21 ± 0.02 ^a^	0.16 ± 0.02 ^bc^	0.00	0.14	0.94
**Bursa of fabricius weight(g)**	3.08 ± 0.19 ^a^	1.22 ± 0.15 ^b^	3.37 ± 0.37 ^a^	1.30 ± 0.11 ^b^	0.00	0.41	0.66
**Bursa of fabricius index (%)**	0.31 ± 0.02 ^a^	0.12 ± 0.01 ^b^	0.34 ± 0.03 ^a^	0.13 ± 0.01 ^b^	0.00	0.32	0.74

CON, control group; CORT, corticosterone-treated group; PB, probiotic-treated group; CORT&PB, CT and PB-treated group. Organ indexes are the ratio of organ weight related to final body weight. Data are expressed as means ± SEM (*n* = 8). Different small letter superscripts a–c in a row indicates significant differences (*p* < 0.05).

## Data Availability

The data presented in this study are available on request from the corresponding authors. The data are not publicly available due to privacy.

## Abbreviations

*ACC*: acetyl-CoA carboxylase; *APOA1*: Apolipoprotein A1; *APOB*: Apolipoprotein B; *CD36*: CD36 molecule; CORT, *DGAT2*: diacylglycerol O-Acyltransferase 2; *FASN*: fatty Acid Synthase; *LPL*: lipoprotein Lipase; *PPAR-α*: peroxisome proliferator activated receptor alpha; *PPAR-γ*: peroxisome proliferator activated receptor gamma; *SCD*: stearoyl-CoA Desaturase; *SREBP1*: sterol regulatory element-binding protein-1; ATGL: Adipose Triglyceride Lipase.
